# Overexpression of DEL-1 Downregulates SH3BP2 Expression and Inhibits *Porphyromonas gingivalis*-induced Gingival Inflammation In Vivo and In Vitro

**DOI:** 10.3290/j.ohpd.b2960781

**Published:** 2022-04-27

**Authors:** Liang Zeng, Fang Ye, Youhong Jin, Yu Luo, Hongshui Zhu

**Affiliations:** a Periodontist, Department of Periodontology, The Affiliated Stomatological Hospital of Nanchang University, The Key Laboratory of Oral Biomedicine, Nanchang, Jiangxi, P.R. China. Idea, hypothesis, experimental design, performed part of the statistical evaluation, proofread the manuscript.; b Periodontist, Department of Periodontology, The Affiliated Stomatological Hospital of Nanchang University, The Key Laboratory of Oral Biomedicine, Nanchang, Jiangxi, P.R. China. Performed part of the experiments, performed part of the statistical evaluation.; c Periodontist, Department of Periodontology, The Affiliated Stomatological Hospital of Nanchang University, The Key Laboratory of Oral Biomedicine, Nanchang, Jiangxi, P.R. China. Performed part of the experiments, performed part of the statistical evaluation.; d Periodontist, Department of Periodontology, The Affiliated Stomatological Hospital of Nanchang University, The Key Laboratory of Oral Biomedicine, Nanchang, Jiangxi, P.R. China. Performed part of the experiments, performed part of the statistical evaluation.; e Physician, VIP Clinic, The Affiliated Stomatological Hospital of Nanchang University, The Key Laboratory of Oral Biomedicine, Nanchang, Jiangxi, P.R. China. Experimental design, interpretation of the results, wrote the manuscript.

**Keywords:** developmental endothelial locus-1, periodontitis, *Porphyromonas gingivalis*, nicotinamide phosphoribosyltransferase, SH3 domain binding protein 2

## Abstract

**Purpose:**

The role of developmental endothelial locus-1 (DEL-1) in *Porphyromonas gingivalis* (*P. gingivalis*)-induced periodontitis and the related molecular mechanisms are unclear. This study aimed to investigate the effect of DEL-1 on SH3 Domain Binding Protein 2 (SH3BP2) expression, and to explore the regulatory role of DEL-1 in periodontal inflammation.

**Materials and Methods:**

We constructed a *P. gingivalis*-induced rat experimental periodontitis model, and cultured *P. gingivalis*-stimulated THP-1 cells in vitro. THP-1 cell viability and cell cycle were examined by CCK-8 and flow cytometry. Rat gingival tissues were collected for hematoxylin-eosin staining. The expression of SH3BP2 and nicotinamide phosphoribosyltransferase (NAMPT) was examined using Western blot.

**Results:**

We found that the proliferation of *P. gingivalis*-infected THP-1 cells was increased by DEL-1. DEL-1 inhibited the expression of NAMPT and SH3BP2 in gingiva tissues of rats with periodontitis as well as in *P. gingivalis*-infected THP-1 cells.

**Conclusions:**

Overexpression of DEL-1 downregulated SH3BP2 expression and reduced gingival inflammation induced by *P. gingivalis*. DEL-1 presents some regulatory effects on gingival inflammation in a *P. gingivalis*-induced rat experimental periodontitis model, suggesting the therapeutic potential of DEL-1 in regulating periodontal inflammation.

Periondontitis is a disruption of the normal function of healthy subgingival plaque biofilm, leading to excessive inflammation and tissue destruction.^6^ It is characterised by the destruction of the bone and gingival tissue supporting the teeth.^27^ About half of the global population over 30 years old have different degrees of periodontitis, and the incidence increases with age.^10^

The pathogenic process of periondontitis involves the reciprocally reinforced interactions between polymicrobial communities and the dysregulated host inflammatory response.^11,14^ In response to infection, circulating neutrophils migrate to the periodontium.^12^ Numerous cytokines that promote leukocyte-endothelial cell interactions have been identified; however, the cytokines that inhibit this process are rarely found.^2,26,34^ Developmental endothelial locus-1 (DEL-1) is a newly discovered cytokine which can suppress the migration of neutrophils to peripheral tissues and promote macrophage efferocytosis.^4,17,29^ A clinical study revealed that expression of DEL-1 was associated with the clinical manifestation of chronic periodontitis.^8^ Aging-associated periodontitis is associated with decreased expression of DEL-1 in mice, and erythromycin inhibits neutrophil infiltration in the periodontium through upregulation of DEL-1.^7,19^ It was found that salivary DEL-1, IL-17, and LFA-1 levels are useful markers for discriminating between periodontal disease, healthy periodontium and gingivitis.^15^ Interleukin (IL)-17 is a pro-inflammatory cytokine that promotes granulocyte production and regulates the recruitment, activation and survival of neutrophils.^3^ IL-17 inhibited DEL-1 expression in gingival endothelial cells, and DEL-1-deficient mice developed spontaneous periodontitis featuring elevated IL-17 expression.^7,18^ In addition, DEL-1 promotes in vitro osteogenesis and blocks inflammatory periodontal bone loss in mice.^30,37^ These findings indicated that DEL-1 is associated with the inflammatory response and osteogenesis in periodontitis.

*Porphyromonas gingivalis* (*P. gingivalis*) is one of the key pathogens involved in periodontal disease.^28^ A rat periodontitis model can be established by infecting rats with *P. gingivalis*, along with ligature use. Gingival inflammatory response and alveolar bone loss were induced in this model.^21^ However, the role of DEL-1 in *P. gingivalis*-induced periodontitis and the related molecular mechanisms are still unclear.

SH3 Domain Binding Protein 2 (SH3BP2) and nicotinamide phosphoribosyltransferase (NAMPT) are downstream genes of the TLR/NF-κB inflammatory signaling pathway, and they are associated with osteoclast formation. SH3BP2 ‘cherubism’ mice showed mandibular bone loss, and TNF-α-dependent autoinflammation.^33^ It has been demonstrated that SH3BP2 could enhance the activity of the TLR/MyD88 signaling pathway, and promote the inflammatory response.^36^ *P. gingivalis* releases a panel of virulence factors to interact with TLRs and trigger immune responses.^16^ Several studies have revealed that NAMPT levels increased in both gingival tissue and gingival crevicular fluid of patients with periodontitis, and the NAMPT level correlated positively with the severity of periodontal disease.^5,24,25^ These reports indicated that SH3BP2 and NAMPT are involved in the pathogenesis of periodontitis, and we speculated that SH3BP2 and NAMPT may play a regulatory role in periodontitis induced by *P. gingivalis*.

Until now, the effect of DEL-1 on the expression of NAMPT and SH3BP2 in macrophages and gingival tissues has not been reported. Research on the therapeutic effect of DEL-1 on periodontitis is still lacking. In this study, we assessed the role of DEL-1 in *P. gingivalis*-induced periodontitis, and the effect of DEL-1 overexpression on SH3BP2 expression was examined in vivo and in vitro. This study may provide novel evidence regarding the effect and the related molecular mechanisms of DEL-1 on gingival inflammation and periodontal disease.

## Materials and Methods

### Cell Culture

THP-1 cells were obtained from Bnbio Company (Beijing, China), and grown in RPMI 1640 Medium (Gibco, Thermo Fisher Scientic; Waltham, MA, USA) supplemented with 10% fetal bovine serum (FBS, HyClone, GE Healthcare Life Sciences; Logan, UT, USA). The cells were maintained at 37°C and 5% CO_2_. The medium was replaced every other day, and the THP-1 cells used for experiments were at passage 5. Wild-type *P. gingivalis* strain ATCC 33277 was purchased from the Guangdong Microbial Culture Center (Guangzhou, Guangdong, China), and cultured in brain heart infusion broth supplemented with defibrillated sheep blood, haemin and vitamin K1 (Qingdao Haina Biotechnology; Qingdao, Shandong, China) at 37°C for 48 h in an anaerobic chamber (85% N_2_, 10% H_2_, and 5% CO_2_). Cells were divided into 4 groups: normal group: THP-1 cells were not infected with *P. gingivalis*; model group: THP-1 cells were infected with *P. gingivalis*; blank group: THP-1 cells were transfected with empty adenoviral vector, then infected with *P. gingivalis*; DEL-1 group: THP-1 cells were transfected with adenoviral expression vector carrying the DEL-1 gene, then infected with *P. gingivalis*. Cells were incubated with *P. gingivalis* for 48 h at 37 °C at a 1:200 cell-to-bacteria ratio.

### Transfection

Adenoviral expression vector carrying the DEL-1 gene and the empty adenoviral vector were provided by Wuhan GeneCreate Biological Engineering (Wuhan, Hubei, China). They were transfected into THP-1 cells using Lipofectamine 2000 (Invitrogen/Thermo Fisher Scientific) according to the manufacturer’s instruction.^38^ After 48 h, the cells were harvested for further experiments.

### Construction of Rat Periodontitis Model

Construction of a rat periodontitis model was carried out according to a previous study^31^ with minor modifications. Twenty male Sprague-Dawley rats (140-180 g, 6 weeks old) were purchased from Hunan Slac Jingda Laboratory Animals (Changsha, Hunan, China). All the animal experiments were approved by the Institutional Animal Care and Use Committee of Nanchang University (approval number: sydwll-2018- 0902; Nanchang, Jiangxi, China) and performed in accordance with the Guide for the Care and Use of Laboratory Animals published by the National Institutes of Health. The rats were randomly divided into 4 groups: normal, model, blank, and DEL-1. The rats were anesthetised by an intraperitoneal injection of 2% pentobarbital sodium (50 mg/kg). 3-0 silk suture was wound around a 0.2 mm orthodontic ligation wire, and then ligated at the neck of the first and second maxillary molars. In the model group, 0.1 ml of *P. gingivalis* (1 x 10^11^ CFU/ml) was injected into the rats’ gingival sulcus between the two maxillary molars once every 2 days for 8 weeks. The periodontitis model was established successfully when bleeding on probing, slight swelling or mild atrophy were observed in rat gingival tissues.^35^ In the blank group, 10 μl of empty adenoviral vector or adenoviral expression vector carrying the DEL-1 gene (DEL-1 group) (1 x 10^9^ plaque-forming units for each vector) was injected into the rats’ gingiva between the first and second maxillary molars for 6 days to induce experimental periodontitis. Rats were sacrificed 18 days after the last injection of adenoviral vector.

### Cell Proliferation Assay

Cell proliferation was determined using a Cell Counting Kit-8 (CCK-8) from KeyGen Biotech (Nanjing, Jiangsu, China) according to the manufacturer’s instructions. Briefly, the cells were incubated with CCK-8 reagent for 2 h at 37 °C, and the absorbance at 450 nm was measured using a FlexStation 3 Multi-Mode Microplate Reader (Molecular Devices; Sunnyvale, CA, USA).

### Cell Cycle Analysis

Cell cycle was examined by FCM with a Cell Cycle Staining Kit (MultiSciences, Hangzhou, Zhejiang, China). The cells were washed once with phosphate-buffered saline (PBS), centrifuged at 1000 rpm for 10 min, and the cell density was then adjusted to 5 x 10^8^-10^9^ cells/l. Cells were fixed in 700 μl absolute ethanol at -20°C for 24 h. After washing with PBS, the cells were incubated with 50 μl Rnase A at 4°C for 30 min, followed by incubation with 50 μl propidium iodide (PI) for 30 min at 37°C. Cells were then harvested and the percentage of cells in each phase of the cell cycle was analysed by NovoCyte Flow Cytometry (ACEA Biosciences; San Diego, CA, USA).

### Real-time Quantitative PCR

Total RNA samples from tissues or cultured cells were extracted using an Ultrapure RNA Extraction Kit (KangWei Biotech; Beijing, China). Briefly, TRIzon and 0.2 ml chloroform were added to the samples, shaken for 15 s, and placed in an ice box for 5 min. After centrifugation at 4°C and 12,000 rpm for 15 min, the aqueous phase was transferred to a new 1.5 ml RNase-free tube, and an equal volume of ethanol (70%, v/v) was added and mixed well by shaking upside down. The mixture was transferred to a collection tube and centrifuged at 4°C and 12,000 rpm for 30 s. The precipitate was subjected to washing with 700 μl RW1 solution and 500 μl RW2 solution, and centrifugation at 4°C at 12,000 rpm for 30 s. The RNA was stored at -80°C. Two μg of total RNA was reverse transcribed to cDNA using a HiFiScript 1st Strand cDNA Synthesis Kit (KangWei Biotech). Gene expression was determined utilising the UltraSYBR Mixture (KangWei Biotech) in 25 μl of reaction volume containing 1 μl of cDNA, 9.5 μl of RNase Free dH20, 12.5 μl of 2 x ULtraSYBR Mixture, 1 μl of sense primer, and 1 μl of antisense primer. Sequences of the primers used were shown in Table 1. The reaction procedures were initial denaturation at 95°C for 10 min, followed by 40 cycles of denaturation at 95°C for 10 s, annealing at 59.5°C for 30 s, and extension at 72°C for 30 s. The relative mRNA level was calculated using the 2(-ΔΔCq) method.

**Table 1:** Quantitative polymerase chain reaction primers used in this study

Gene	Primer sequence (5’-3’)	Amplicon size (bp)
DEL-1		171
Sense	TTGGCTGATGGTTCCTTTT	
Antisense	CCCTCGGTATGCTTCACTTA	
SH3BP2		128
Sense	ATGGGCAGAGTTTCAGGAGC	
Antisense	GGTGTTGACGAAGACCGAGTT	
NAMPT		208
Sense	CCGACTCCTACAAGGTTACTCA	
Antisense	TTGGCTTCCTGGATTTTCTC	
GAPDH		106
Sense	CAATGACCCCTTCATTGACC	
Antisense	GAGAAGCTTCCCGTTCTCAG	

SH3BP2, SH3 Domain Binding Protein 2; NAMPT, nicotinamide phosphoribosyltransferase.

### Western Blot

The tissue and cell lysates for Western blot analysis were prepared in RIPA buffer (Sigma-Aldrich) with Protease Inhibitor Cocktail (Sigma-Aldrich). The protein samples were separated by 10% SDS-PAGE, and transferred onto polyvinylidene fluoride membranes (Merck Millipore; Billerica, MA,USA). After blocking with 5% non-fat milk in TBST for 1 h, proteins were probed with specific antibodies at 4°C overnight, and with horseradish peroxidase-conjugated secondary antibody at 37°C for 1 h. Signals were visualised by enhanced chemiluminescence substrate kit (GE Healthcare; Piscataway, NJ, USA). The primary antibodies used were as follows: Rabbit Polyclonal Anti-SH3BP2 (1/1000; Beijing Boisynthesis Biotechnology; Beijing, China), Rabbit Polyclonal Anti-NAMPT (1/1000; Affinity Biologicals; Ancaster, ON, Canada) and Mouse Monoclonal Anti-GAPDH (1/2000, ZSBIO; Beijing, China).

### HE Staining

The rat gingival tissues were fixed in 10% paraformaldehyde, then embedded in paraffin. After deparaffinisation and rehydration, the sections (4 mm) were stained with hematoxylin for 5 min and eosin for 3 min. The slides were examined under a microscope (Nikon, Japan).

### Statistical Analysis

Statistical analysis was performed with SPSS 19.0 software (SPSS; Chicago, IL, USA). All the data were presented as the means ± standard deviations from at least three separate experiments. Differences between groups were compared using one-way ANOVA followed by the least significant differences (LSD) post-hoc test. p < 0.05 was considered statistically significant.

## Results

### Effect of DEL-1 on Cell Growth of THP-1 Cells Infected with *P. gingivalis*

**Fig 1:**
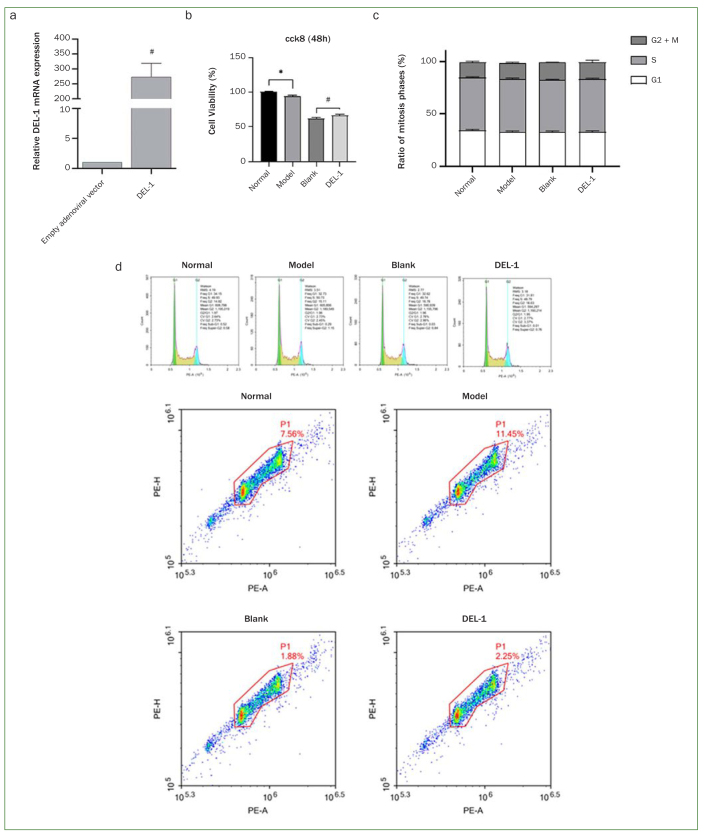
Effect of DEL-1 on cell growth of THP-1 cells infected with *P. gingivalis*. (a) The relative mRNA expression of DEL-1 in THP-1 cells transfected with empty adenoviral vector or adenoviral expression vector carrying the DEL-1 gene. #p < 0.01. (b) Effect of DEL-1 on cell viability of THP-1 cells infected with *P. gingivalis*. (c, d) Effect of DEL-1 on cell cycle of THP-1 cells infected with *P. gingivalis*. The distribution of cells in the gate was dumbbell-shaped. The right part of dumbbell consists of G2/M phase cells, and the middle part of S phase cells. n = 9 independent samples. *p < 0.05, model vs normal; #p < 0.05, DEL-1 vs blank. DEL-1: developmental endothelial locus-1.

Empty adenoviral vector and adenoviral expression vector carrying the DEL-1 gene were transfected into THP-1 cells, and the results from real-time quantitative PCR analysis demonstrated that the relative mRNA expression of DEL-1 was statistically significantly higher in the DEL-1 group compared with the empty adenoviral vector group (Fig 1a). Cell viability was assessed by CCK-8, and the results indicated that *P. gingivalis* significantly suppressed cell viability. DEL-1 overexpression could promote cell viability of THP-1 cells infected with *P. gingivalis* (Fig 1b). The cell cycle was assessed by FCM, and we found no statistically significant difference in the cell mitotic cycle between these groups (Figs 1c and 1d).

### Effect of DEL-1 on NAMPT and SH3BP2 Protein Expression in THP-1 Cells Infected with *P. gingivalis*

**Fig 2:**
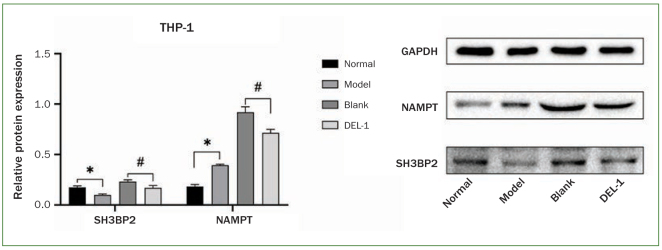
Effect of DEL-1 on NAMPT and SH3BP2 protein expression in THP-1 cells infected with *P. gingivalis*. n = 9 independent samples. *p < 0.05, model vs normal; #p < 0.05, DEL-1 vs blank. DEL-1: developmental endothelial locus-1; NAMPT: nicotinamide phosphoribosyltransferase; SH3BP2: SH3 domain binding protein 2.

Using Western blot, we examined the effect of DEL-1 on NAMPT and SH3BP2 protein expression in THP-1 cells infected with *P. gingivalis*. Results showed that *P. gingivalis* induced NAMPT protein expression in THP-1 cells. DEL-1 overexpression downregulated the *P. gingivalis*-stimulated NAMPT protein expression level. The SH3BP2 protein expression level decreased statistically significantly by *P. gingivalis* infection. DEL-1 overexpression downregulated SH3BP2 protein expression in *P. gingivalis*-infected THP-1 cells (Fig 2).

### Effect of DEL-1 on *P. gingivalis*-induced Periodontitis in a Rat Model

**Fig 3:**
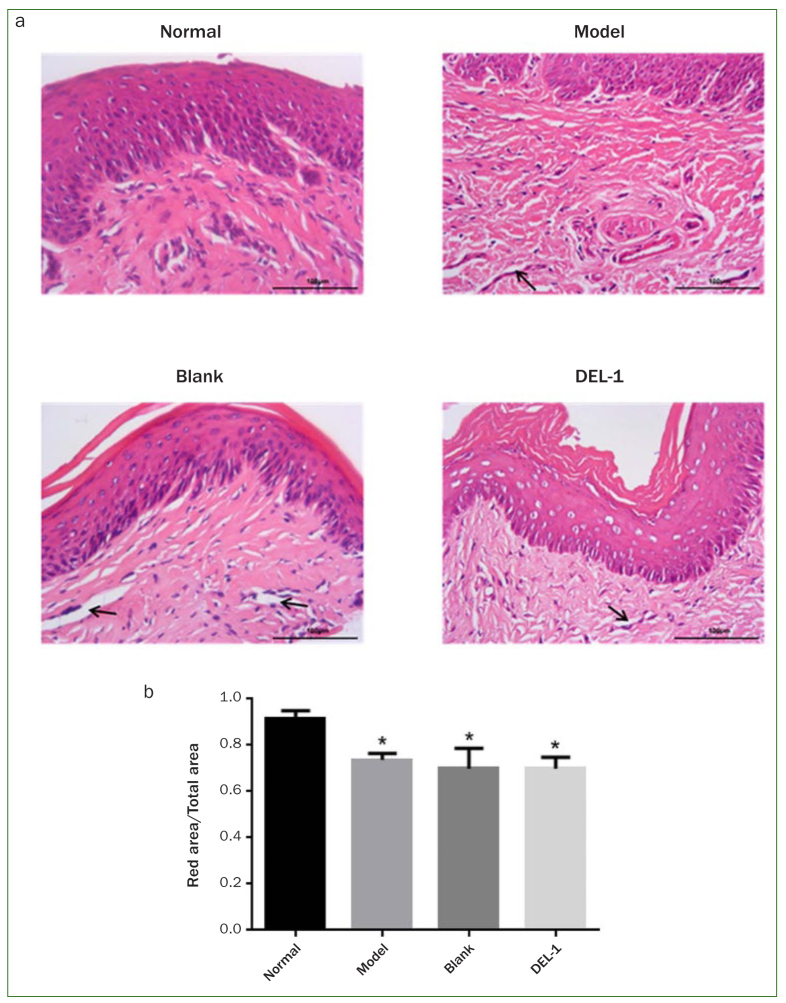
HE staining of gingival tissues in a rat periodontitis model. Arrow: the widening of periodontal ligament space. Magnification 400X. HE: hematoxylin-eosin.

Subsequently, we examined the effects of DEL-1 on *P. gingivalis*-induced periodontitis in a rat model. The rat gingival tissues were collected for HE staining. As shown in Fig 3a, compared with the normal rats, the connective tissues of rat gingiva were looser in rats infected with *P. gingivalis*, indicating that these *P. gingivalis*-infected rats had swollen gums. The model, blank and DEL-1 groups showed obvious oedema and hyperplasia of fibrous connective tissue, the degeneration and degradation of periodontal ligament collagen, and widened periodontal ligament space. It seems that there was no statistically significant difference between the model, blank and DEL-1 groups. An area of periodontal ligament was selected, and the ratio of the red-stained area to the total selected area was calculated. As shown in Fig 3b, the proportion of red area in model group, blank group and DEL-1 group was lower than that in the normal group, indicating that periodontal ligament tissue was looser in rats infected with *P. gingivalis*. There was no significant difference between the model, blank and DEL-1 groups.

### Effect of DEL-1 on NAMPT and SH3BP2 Protein Expression in a Rat Periodontitis Model

**Fig 4:**
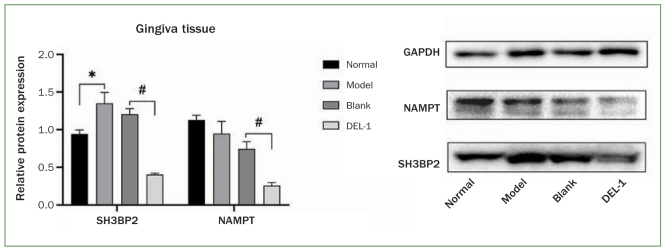
Effect of DEL-1 on NAMPT and SH3BP2 protein expression in a rat periodontitis model. *p < 0.05, model vs normal; #p < 0.05, DEL-1 vs blank. DEL-1: developmental endothelial locus-1; NAMPT: nicotinamide phosphoribosyltransferase; SH3BP2: SH3 domain binding protein 2.

We collected rat gingival tissues to investigate the effect of DEL-1 on NAMPT and SH3BP2 protein expression. As shown in Fig 4, the SH3BP2 protein level increased statistically significantly in the model group compared with that in the control group; however, we did not find a statistically significant difference in NAMPT protein level between the two groups. Compared with rats injected with empty adenoviral vector, the protein levels of NAMPT and SH3BP2 were statistically significantly downregulated in rats injected with adenoviral expression vector carrying the DEL-1 gene.

## Discussion

In this study, we found that overexpression of DEL-1 downregulated NAMPT and SH3BP2 in gingiva tissues of rats with periodontitis as well as in *P. gingivalis*-infected THP-1 cells. It is speculated that DEL-1 can inhibit gingival inflammation and alveolar bone damage, which shows the potential application value of DEL-1 in the treatment of periodontitis.

*P. gingivalis* can reduce the host’s non-specific immune response, and evade the attack of immune cells. On the other hand, it can induce gingival inflammatory responses, and absorb nutrients from the gingival crevicular fluid containing inflammatory exudates and blood.^1,13^ In the present study, *P. gingivalis*-stimulated THP-1 cells were cultured in vitro. We found that THP-1 cell viability was inhibited by *P. gingivalis*, and the expression of NAMPT protein was upregulated by *P. gingivalis*. SH3BP2 protein expression was statistically significantly downregulated by *P. gingivalis*. SH3BP2 protein degradation is an important regulatory pathway for its function,^23^ and protein expression level is valuable in evaluating SH3BP2 function. The results from this study indicated that *P. gingivalis* inhibited the function of SH3BP2 in THP-1 cells, and this finding is in accordance with the fact that *P. gingivalis* can evade the host immune response to some extent.^1^ Further, we found that DEL-1 inhibits the expression of NAMPT and SH3BP2 in *P. gingivalis*-infected THP-1 cells, and cell viability of THP-1 cells was increased by DEL-1. Increased cell viability of monocytes can promote the rapid production and regression of proinflammatory factors and NO, which helps to optimise the innate immune response. These results suggested that DEL-1 may inhibit the inflammatory response of THP-1 cells. The in vivo experiments revealed that SH3BP2 expression statistically significantly increased in the gingival tissues of rats with periodontitis, and DEL-1 could inhibit SH3BP2 and NAMPT expression. This is related to the ability of *P. gingivalis* to escape the host immune response and amplify destructive inflammation, and DEL-1 inhibits inflammation. Collectively, these results suggested that DEL-1 has a regulatory effect on periodontitis; however, it is difficult to judge whether the regulatory role is beneficial to patients with periodontitis. DEL-1 could suppress the inflammatory response, indicating that DEL-1 might prevent immune cells from attacking bacteria; on the other hand, DEL-1 could reduce the damage an inflammatory reaction has on tissue, and prevent *P. gingivalis* from obtaining nutrients in inflammatory exudate. Further studies should assess the regulatory mechanism of DEL-1 on periodontitis, and explore the strategy of using DEL-1 to treat periodontitis.

NAMPT is a cytokine involved in the regulation of inflammatory responses.^9,32^ In vitro studies demonstrated that NAMPT modulates the actions of enamel matrix derivative, and may compromise the regenerative capacity of periodontal ligament cells.^20^ Besides its pro-inflammatory function in gingival tissue, it was shown that NAMPT can upregulate the expression and the activities of MMP1, MMP3 and RANKL in human gingival fibroblasts, resulting in alveolar bone loss.^22^ These reports indicated that NAMPT is an adverse factor in periodontitis. Interestingly, the present study found the NAMPT protein level unaltered in the gingival tissues of rats with periodontitis. This may be due to the fact that NAMPT has the dual functions of phosphoribosyltransferase and cytokine. In addition, although NAMPT could be induced by *P. gingivalis* in periodontitis, *P. gingivalis* was aggregated in a small area of gingival tissue. Therefore, the change of NAMPT protein expression was subtle. The effect of *P. gingivalis* on the expression of SH3BP seems to be inconsistent in THP-1 cells and in rats; it is mainly attributed to the fact that *P. gingivalis* can trigger the inflammatory reaction, but it also can inhibit the inflammatory reaction of surrounding tissues to escape from attack by immune cells.^1,35^

Several limitations associated with the present study should be mentioned. First, this study mainly investigated *P. gingivalis*-induced gingival inflammation; periodontal bone loss was not evaluated. Second, we did not examine the correlation between overexpression of DEL-1 and the biological effects. More comprehensive parameters need to be tested, and their correlation with DEL-1 needs to be examined in future studies.

## Conclusion

This study demonstrated that DEL-1 has some regulatory effects on gingival inflammation in a *P. gingivalis*-induced rat experimental periodontitis model. Overexpression of DEL-1 downregulated SH3BP2 expression and reduced gingival inflammation induced by *P. gingivalis*. These findings suggest the therapeutic potential of DEL-1 in regulating periodontal inflammation.
